# Revisiting the Role of Plant Transcription Factors in the Battle against Abiotic Stress

**DOI:** 10.3390/ijms19061634

**Published:** 2018-05-31

**Authors:** Sardar-Ali Khan, Meng-Zhan Li, Suo-Min Wang, Hong-Ju Yin

**Affiliations:** State Key Laboratory of Grassland Agro-ecosystems, College of Pastoral Agriculture Science and Technology, Lanzhou University, Lanzhou 730000, China; ali.khan13@lzu.edu.cn (S.-A.K.); limz11@lzu.edu.cn (M.-Z.L.); smwang@lzu.edu.cn (S.-M.W.)

**Keywords:** abiotic stress, gene expression, transcription factors, stress response

## Abstract

Owing to diverse abiotic stresses and global climate deterioration, the agricultural production worldwide is suffering serious losses. Breeding stress-resilient crops with higher quality and yield against multiple environmental stresses via application of transgenic technologies is currently the most promising approach. Deciphering molecular principles and mining stress-associate genes that govern plant responses against abiotic stresses is one of the prerequisites to develop stress-resistant crop varieties. As molecular switches in controlling stress-responsive genes expression, transcription factors (TFs) play crucial roles in regulating various abiotic stress responses. Hence, functional analysis of TFs and their interaction partners during abiotic stresses is crucial to perceive their role in diverse signaling cascades that many researchers have continued to undertake. Here, we review current developments in understanding TFs, with particular emphasis on their functions in orchestrating plant abiotic stress responses. Further, we discuss novel molecular mechanisms of their action under abiotic stress conditions. This will provide valuable information for understanding regulatory mechanisms to engineer stress-tolerant crops.

## 1. Introduction

The urgent demand for sustainable food production is still far from realization due to an alarming expansion of human population, along with uncertain threats linked with global climate deterioration, increasing soil salinization, and freshwater scarcity. Presently, approximately 800 million people are undernourished worldwide [1]. To feed the 9.6 billion people projected by 2050, an additional 70% increase in agricultural productivity is needed [2,3]. In nature, crop growth and productivity are adversely affected by various abiotic stresses, including extreme temperatures, salinity, drought, and other adverse conditions. Besides, crops suffering from abiotic stresses are generally more susceptible to biotic stress factors, such as pathogens, insects, and weeds, which increase the losses considerably. Together, these environmental constraints lead to more than 50% average yield losses for major crops globally [4,5]. Therefore, breeding stress-tolerant cultivars against multiple environmental stresses, with higher quality and yield, are required to match the food demands of the burgeoning human population [2]. Since the multigenic nature of abiotic stress traits and the narrow genetic pool have resulted in the limited success of traditional breeding, the use of transgenic technologies to breed stress-tolerant cultivars is currently becoming popular. Understanding the molecular principles and mining the stress-responsive genes that govern plant responses against abiotic stress factors is one of the prerequisites to develop stress-resistant crop varieties.

**Figure 1:**
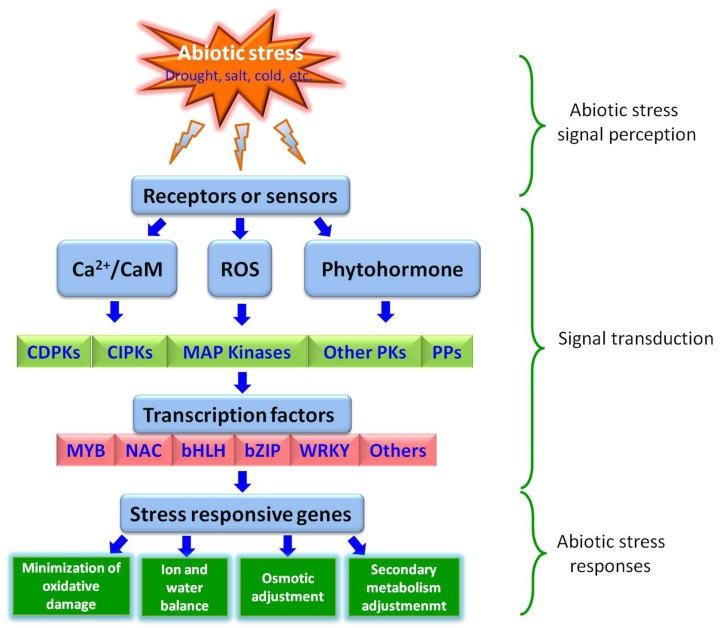
Model for transcription factors regulating abiotic stress-signaling pathways.

Being a sessile organism in nature, plants have evolved various intricate acclimatization strategies to combat unfavorable environmental conditions [6]. At the biochemical, physiological, and cellular levels, the production of phenylpropanoid-derived compounds and phytohormones, synthesis of antioxidants, cuticular wax, and osmolytes, adjustments of the membrane system, inhibition of cell growth and proliferation, and closure of stomata are associated with plant adaptive responses against abiotic stresses [7,8,9,10,11]. Similarly, at the molecular level, the stress-signaling pathways play key roles in plant abiotic stress response via linking the sensing mechanism and the genetic response. Generally, a stress signal transduction pathway comprises the following key steps: (1) signal perception; (2) signal transduction; and (3) stress response (Figure 1).

The first step in activation of a signaling cascade for any given abiotic stress is the recognition of stress signal via receptors located on the membrane of the plant cell. Recently, a body of researchers showed that various plasma membrane proteins, like COLD1 (CHILLING-TOLERANCE DIVERGENCE 1) [12], OSCA1 (reduced hyperosmolality-induced calcium increase1) [13], MSLs (MscS-like proteins) [14], CNGCs (cyclic nucleotide-gated channels), GLR (glutamate receptor-like) channels [15], histidine kinases, calcium channels (responsible for Ca fluxes), and GPCRs (G-protein-coupled receptors) [16] are the putative sensors for abiotic stress signals (Figure 1). After recognition, these sensors transmit the signal downstream through phytohormones and second messengers such as Ca^2+^ and ROS (reactive oxygen species) [17] (Figure 1). The second messengers, like ROS, trigger the activation of another set of ROS-modulated protein kinases (PKs) and protein phosphatases (PPs), including MAPK (mitogen-activated protein kinase) cascades, CDPKs (calcium-dependent protein kinases), CBLs (calcineurin-B-like proteins), CIPK (CBL-interacting protein kinase), and many other PKs, as well as PPs such as some PP2Cs (protein phosphatase 2Cs) (Figure 1). Subsequently, these PKs and PPs deliver the information downstream and trigger a series of phosphorylation/dephosphorylation cascades, especially the phosphorylation/dephosphorylation of transcription factors (TFs) [18,19], that finally culminates either directly in the expression of functional genes involved in cellular protection, or indirectly in the expression of regulatory genes participating in signaling cascades and transcriptional regulation of gene expression [20,21] (Figure 1). The expressions of all these stress-responsive genes mentioned above are regulated by TFs activated via phosphorylation/dephosphorylation. Contrary to functional genes, a single TF can regulate a cluster of downstream target genes. These abilities allow them to be excellent candidate genes for genetic manipulation of complex stress tolerance traits [22,23].

To date, based on genome wide analysis, a great deal of TFs belonging to different families, such as MYB, bHLH, WRKY, bZIP, NAC, and so on, have been identified in different plant species [24,25,26,27]. Interestingly, functional analysis of a wide range of TFs via knockout/knockdown mutants and overexpression transgenic lines in model plants, as well as in different crops, have reported the involvement of TFs in the regulation of abiotic stress responses, such as in drought, salt, and cold tolerance (Table 1). In the present review, we specifically highlight the current developments in understanding TFs, with particular emphasis on their functions in orchestrating plant abiotic stress responses, and discuss novel molecular mechanisms of their action under abiotic stress conditions.

## 2. Transcription Factors Involved in Abiotic Stress Responses

### 2.1. MYB Transcription Factors

MYB (v-myb avian myeloblastosis viral oncogene homolog) proteins represent one of the largest TF families in plants, characterized by a highly conserved MYB domain (DNA-binding domain) at the N-terminus [94]. Based on the number of adjacent repeats in the MYB domain, MYB proteins are classified into four subfamilies, including R1-MYB, R2R3-MYB, 3R-MYB, and 4R-MYB, with 1, 2, 3, and 4 peptide repeats, respectively [95]. Among these subfamilies, R2R3-MYB is the largest subfamily, with 126 and 109 members in Arabidopsis and rice, respectively [25].

MYB TFs have been found to play a crucial role in the transcriptional control of numerous physiological and biochemical processes, including plant development, cell fate determination, and secondary metabolism [96]. In addition, MYB proteins are well documented for their involvement in mediating abiotic stress responses in many model plants and crop species [28,97,98]. For example, overexpression of Arabidopsis *MYB96* TF in *Camelina sativa* promoted drought resistance, potentially through the accumulation of cuticular wax [99]. Recently, a study with Arabidopsis showed that this gene (*MYB96*) can be transiently induced by cold stress and ensures plant freezing tolerance via integration of cold and ABA (abscisic acid) signaling pathways [29]. Also, the expression level of *AtMYB74* was dramatically increased under NaCl treatments and was shown to be transcriptionally regulated by the RdDM (RNA-directed DNA methylation) pathway via repressing the accumulation of 24-nt siRNAs under salt stress [100]. Likewise, knockdown of *AtMYB14* by artificial microRNA increases cold stress tolerance via affecting CBF (C-repeat binding factor) genes, the knockdown approach strongly suggests an important role of *AtMYB14* in low-temperature tolerance [101]. Besides, salt-related *MYB1* (*SRM1*) negatively regulates seed germination and vegetative growth in Arabidopsis under salt stress [102]. In soybean, transcripts of *GmMYB84* were induced by salt stress, drought, ABA, and H_2_O_2_. Overexpression of *GmMYB84* in soybean resulted in longer primary roots, high survival rates, and a lower dehydration rate under drought stress [30]. Additionally, *GmMYB12B2* was drastically induced by NaCl treatment and UV irradiation, but not by drought, cold, and ABA stresses. Constitutive expression of *GmMYB12B2* in Arabidopsis facilitated tolerance against UV radiation and salt treatment [31]. In rice, overexpression of a novel MYB-related protein, *OsMYBR1*, displayed greater tolerance to dehydration stress and reduced sensitivity to ABA in terms of higher soluble sugar and free proline levels [103]. Similarly, in maize (*Zea mays*), overexpression of *OsMYB55* led to increased plant biomass and reduced leaf damage associated with exposure to high temperature and drought, possibly due to increased expression of stress-responsive genes [32]. In addition, a novel MYB protein, *OsMYBc*, was shown to regulate both the expression and role of *OsHKT1;1* in controlling Na^+^ concentration, which in turn prevents sodium toxicity in leaf blades of rice [33]. Using microarray data, Huang et al. [104] identified *OsMYB511* from rice seedlings and revealed that this gene is an early regulator of cold stress response in rice, supported by dramatic induction of *OsMYB511* under cold stress, and its high expression during the early developmental stage in panicles, interestingly accustomed to circadian rhythm regulation. Similarly, *MID1* [34], *OsMYB48-1* [105], and *OsMYB91* [35] TF genes were reported to participate in salt and/or drought stress tolerance of rice. Moreover, a set of MYB TFs isolated from wheat (*Triticum aestivum*) has been shown to mediate abiotic stress responses. According to a more recent report, transgenic plants with overexpressed wheat *TaSIM* gene demonstrated significantly longer roots in addition to increased expression levels of genes (*RD22*, *RD29A*) linked to both ABA-dependent and ABA-independent signaling [36]. Likewise, transcription levels of *TaMYB31* and *TaMYB74* were upregulated in response to drought stress in wheat [106], while the increased dehydration tolerance in *TaMYBsm1-D*-harboring Arabidopsis lines was attributed to the high production of proline and poor malondialdehyde (MDA) content, as well as lower water loss rates [107]. Li et al. [24] found that *FtMYB9* is rapidly induced in tartary buckwheat (*Fagopyrum tataricum*) on exposure to drought, salt, ABA, and cold treatments at the seedling stage. Elevated salt and drought resistances were obtained by introduction of *FtMYB9* in Arabidopsis under the control of CaMV 35S promoter, but, interestingly, the transgenic plants were highly sensitive to ABA at seed germination and seedling stages, accompanied by enhanced salt and drought tolerance. Nevertheless, the precise mechanism needs further investigation. Recently, *Medicago truncatula* MtMYBS1 TF was found to be able to boost drought and salt tolerance in Arabidopsis via a significant increase in primary root growth [37]. Likewise, two other MYB proteins, MtMYB3 and MtMYB61 from *M. truncatula* are involved in mediating cold tolerance [108]. Also, in tomato (*Solanum lycopersicum*), an MYB TF, SlAN2 positively regulates anthocyanin biosynthesis in vegetative tissues under high light and cold conditions [109]. Campos et al. [38] identified a salt-sensitive phenotype of tomato *ars1* mutant, resulting from a single T-DNA insertion in the *ARS1* gene, encoding an R1-MYB TF. Under salt acclimation, the mutant was able to accumulate high Na^+^ in the leaves followed by a decrease in stomatal conductance and lower transpiration rate, indicating that the *ARS1* gene contributes to stomatal movement under salt stress. In addition, ectopic expression of the cotton (*Gossypium arboreum*) *GaMYB62L* gene in Arabidopsis developed better drought resistance [39]. Similarly, *GaMYB85* also promotes drought tolerance in transgenic Arabidopsis by elevating chlorophyll and free proline content, with a subsequent rise in relative water content [40]. Likewise, transgenic analysis of *MdoMYB121* revealed that the enhanced tolerance against multiple abiotic stresses results from over accumulation of osmoprotectants in *MdoMYB121*-harboring apple (*Malus pumila*) and tomato plants [110]. Besides, a very recent study identified two R2R3 MYB proteins, MYB88 and MYB124 involved in promoting H_2_O_2_ detoxification and anthocyanin accumulation in apple under cold stress, suggesting their role in mediating cold tolerance [41]. Similarly, Fang et al. [111] also reported that the poplar (*Populus tremula*) *PtrSSR1* gene, a member of R2R3 MYB family, is involved in elevating endogenous ABA content that leads to faster induction of lateral root emergence in transgenic Arabidopsis lines under salt stress, indicating that *PtrSSR1* correlates with the regulation of LRE and ABA signaling to impart salt tolerance. In transgenic Arabidopsis, the decreased osmotic potential and elevated levels of peroxidase and proline content in response to salt stress were attributed to overexpression of the *PacMYBA* gene from sweet cherry (*Prunus avium*) [42]. Additionally, *CiMYB5* and *CiMYB3* cloned from chicory (*Cichorium intybus*) were shown to be involved in the degradation of the fructan pathway in response to abiotic stress [112]. Sun et al. [113] demonstrated that the *Poncirus trifoliata PtsrMYB* protein participates in dehydration tolerance, probably by modulation of polyamine synthesis due to regulation of the *ADC* (arginine decarboxylase) gene. Similarly, *LpMYB1*, cloned from *Lablab purpureus*, also mediates drought stress tolerance [114]. In addition, overexpression of *ChiMYB*, isolated from *Chrysanthemum indicum*, resulted in higher survival rates in Arabidopsis under salt stress [115]. Likewise, overexpression of the *Jatropha curcas* gene, *JcMYB2*, enhanced salt and cold tolerance of transgenic Arabidopsis [116]. Overexpression of *MpMYBS3* in banana (*Musa paradisiaca*) conferred significantly higher cold tolerance [117]. On the contrary, transgenic tomato harboring the anthocyanin-associated R2R3-MYB TF, LeAN2, showed enhanced thermotolerance [118].

### 2.2. bHLH (Basic Helix-Loop-Helix) Transcription Factors

The bHLH (basic helix-loop-helix) transcription factors constitute the second largest family in angiosperms and are universally distributed in all eukaryotic organisms [43]. The highly conserved bHLH signature domain comprises two distinct functional regions, a basic N-terminal DNA-binding region, and a helix-loop-helix region involved in protein–protein interactions via the formation of homo- and heterodimeric complexes located at the C-terminal end, which is a prerequisite for TF function [119,120]. bHLH proteins form a monophyletic cluster and regulate gene expression in plants by recognizing and binding to the E-box (5′-CANNTG-30-3′) and G-box (5′-CACGTG-3′) consensus core DNA motifs present in the promoters of its target genes [121,122]. Since the first report of a bHLH TF gene in maize (regulatory gene *R*) [122], a considerable number of bHLH TFs have been reported in different plant species.

To date, functional analysis has revealed that bHLH transcription factors play critical roles in a myriad of biological processes, such as plant development [123], flavonoids biosynthesis [124], flowering [125], and photosynthesis [44]. Similarly, accumulating evidence has indicated that bHLH TFs also participate in regulating various abiotic stresses. For example, the expression of *AtbHLH68*, a putative non-DNA-binding bHLH gene, was altered in Arabidopsis in an organ-specific manner with exogenous application of ABA, and its overexpression conferred transgenic plants significant drought resistance, likely via regulation of ABA homeostasis [43]. Further, *AtbHLH112* overexpressors displayed better ROS-scavenging abilities, as well as elevated levels of proline that led to high resistance against multiple abiotic stresses, including salt and drought [45]. In another study, transcripts of *bHLH122* were shown to be strongly induced by osmotic, drought and salt stresses, but not by ABA. In contrast to the *bhlh122* loss-of-function mutant, *bHLH122* overexpressing lines were more resistant to osmotic, drought, and salt stress. Interestingly, *bHLH122* can significantly enhance cellular ABA levels via directly repressing the expression of *CYP707A3*, a key ABA 8′-hydroxylase required for ABA catabolism. The data suggest that *bHLH122* is positively involved in regulating abiotic stress tolerance in addition to repression of ABA catabolism [21]. Moreover, Babitha et al. [121] reported that co-overexpression of *AtbHLH17* with *AtWRKY28* in Arabidopsis, promotes abiotic stress tolerance in terms of longer roots and superior plant growth in response to mannitol and desiccation stresses, respectively. In Arabidopsis, higher accumulation of flavonoids due to overexpression of *AmDEL*, a bHLH TF, was reported to be a positive response against salt and drought stress [126]. Functional characterization of *OsbHLH068* and its homologous gene, *AtbHLH112*, revealed that, while both these genes have opposite roles in controlling flowering phenotypes, they share partially redundant functions to confer salt stress tolerance, as shown by prolonged root length and lower accumulation of H_2_O_2_ under salt stress [45]. In wheat, gene expression analysis showed that *TabHLH39* is induced in response to salt, polyethylene glycol, and cold, and exhibits differential expression patterns in roots, stems, and leaves. Transgenic Arabidopsis with overexpressed *TabHLH39* displayed significant resistance to multiple abiotic stress conditions during the seedling stage via altered physiological indices [47]. Very recently, *FtbHLH2*, cloned from Tartary buckwheat was reported to elevate cold tolerance, as observed with higher photosynthetic efficiency and lower reactive oxygen species (ROS) in transgenic plants under cold stress [48]. Likewise, the improved drought/oxidative stress resistance of *FtbHLH3*-overexpressing Arabidopsis lines was attributed to lower MDA and higher activation of the antioxidant system than the wild type (WT) [49]. In addition, the tomato Hoffman’s anthocyaninless gene, *AH*, encoding a bHLH TF, is involved in enhancing low-temperature tolerance in tomato seedlings via accumulating a greater amount of anthocyanin and regulating abiotic stimuli genes [50]. Similarly, ectopic expression of *PebHLH35* from *Populus euphratica* provided tolerance against water-deficit stress through changes in several anatomical and physiological parameters, such as stomatal aperture, stomatal density, and photosynthetic and transpiration rates [44]. Similarly, the stress-induced *PtrbHLH* from *P. trifoliate* [127], *MabHLH1*/*2*/*4* from banana [128], *PubHLH1* from pear (*Pyrus ussuriensis*) [51], and *ThbHLH1* from *Tamarix hispida* [52] have been shown to enhance tolerance against abiotic stresses. Additionally, some bHLH TFs involved in regulating element-deficiency and iron stress responses have been reported. For instance, *TabHLH1* overexpressing plants displayed improved tolerance against N and Pi starvation via transcriptional regulation of nutrient transporter genes *NtPT1* and *NtNRT2.2* [53]. Besides, transgenic plants overexpressing Arabidopsis *bHLH34* and *bHLH104* demonstrated increased expression of iron (Fe) deficiency-responsive genes and Fe accumulation. By contrast, loss-of-function of *bHLH34* and *bHLH104* resulted in the reduction of Fe content and disruption of Fe deficiency response [129]. Similarly, using multiple knockout mutants, Wang et al. [130] reported that, in Arabidopsis, *bHLH38*, *bHLH39*, *bHLH100*, and *bHLH101* play vital roles under limiting iron conditions and are required for activation of Fe deficiency responses and uptake. Moreover, functional analysis of the triple knockout mutant *bhlh39 bhlh100 bhlh101* revealed that these TFs act in the iron intake and internal metabolic responses to Fe deficiency [131]. Also, *CmbHLH1* from chrysanthemum (*Chrysanthemum morifolium*) regulates iron uptake via H^+^-ATPase-mediated acidification of the rhizosphere under Fe deficiency [132]. Likewise, in *Populus tomentosa*, *PtFIT* is involved in conferring Fe deficiency tolerance, as indicated by elevated chlorophyll content and Chl a/b ratio in overexpressing transgenic plants [54].

### 2.3. WRKY Transcription Factors

WRKY proteins cover a large group of transcription factors that can be found throughout the green plants. The common feature of WRKY proteins is the presence of an approximately 60-amino-acid DNA-binding domain, known as the WRKY domain, followed by a zinc-finger motif at the C-terminus [133,134]. WRKY family proteins are categorized into three distinct groups (I, II, and III) according to the number of WRKY domains and zinc-finger motifs [135]. Most WRKY proteins were found to recognize and bind specifically to W-box (TTGACT/C), a consensus cis-element commonly found in the promoters of many defense and abiotic stress response-related genes, implying that they play important roles in plant biotic and abiotic stress tolerance [135,136].

Recent knowledge has shown that WRKY proteins participate in various abiotic stresses in different plant species [55,137]. For example, WRKY46, WRKY54, and WRKY70 were found to cooperate with BES1 TF in Arabidopsis to promote brassinosteroid (BR)-regulated plant growth, but negatively regulate dehydration tolerance [138]. Salinity-inducible *WRKY71* has been shown to hasten flowering in Arabidopsis under salt stress, thereby helping the plant to complete its life cycle earlier in order to escape salt stress [56]. Sun et al. [57] observed that activated expression of *AtWRKY53*, a member of group III WRKY proteins, can modulate stomatal movement via boosting starch metabolism that facilitates stomatal opening, and by reducing H_2_O_2_ content in guard cells, thus negatively participating in regulating dehydration tolerance. Further, heterologous expression of maize *ZmWRKY17* in Arabidopsis resulted in reduced ABA sensitivity—as indicated by healthy green cotyledons and longer roots—in response to exogenous ABA application nevertheless increased plant sensitivity to NaCl stress [58]. Similarly, in rice, *OsWRKY30* and *OsWRKY47* have been reported to participate in conferring drought stress tolerance. Meanwhile, *OsWRKY71* functions as a positive regulator of cold tolerance by regulating the expression of downstream target genes, such as *OsTGFR* and *WSI76* [55,59,139]. In a similar manner, a rice transcriptional repressor, *WRKY76*, not only mediates cold stress tolerance but also has a function in plant defense against blast disease through enhanced expression of abiotic stress-related genes and suppression of PR genes, respectively [136]. *TaWRKY146* cloned from wheat exhibited significant expression in the leaves and roots of wheat seedlings following osmotic stress and rendered drought tolerance to transgenic Arabidopsis via facilitating stomatal closure [60]. Similarly, the enhanced drought and salt tolerance observed from *TaWRKY10* transgenic tobacco was attributed to a reduction in ROS accumulation and regulation of osmotic balance [140]. Additionally, *TaWRKY93* improved abiotic stress tolerance in overexpressing transgenic Arabidopsis, as indicated by longer primary roots and more proline content compared to WT [137]. In soybean, the genetic analysis demonstrated that *GmWRKY27* enhances salt and drought tolerance, supported by measurements of proline and ROS content [61]. Besides, a recent study uncovered that overexpression of tomato SlWRKY3 protein promotes physiological indices associated with photosynthesis, higher accumulation of K^+^ and Ca^2+^ in the leaves, and reduced sodium and proline content [62]. Likewise, *SlWRKY39* and *SlDRW1* conferred both biotic and abiotic stress tolerance to tomato via activating the expression of both stress and pathogenesis-related genes [141,142]. In transgenic *M. truncatula*, *MtWRKY76* interacts with the ASR protein, as well as triggers the induction of abiotic stress-responsive genes, resulting in improved drought and salt tolerance [63]. Similarly, a *GhWRKY6*-like gene from cotton (*Gossypium hirsutum*) was found to be involved in scavenging reactive oxygen species and activating the ABA signaling pathway, thereby enhancing salt tolerance in Arabidopsis. By contrast, silencing of the *GhWRKY6*-like gene through VIGS (virus-induced gene silencing) in cotton increased abiotic stress sensitivity [64]. Equally, two other WRKY genes from cotton, *GhWRKY41* and *GhWRKY68*, positively regulate salt and drought stress tolerance via affecting numerous physiological indices, such as stomatal closure and ROS accumulation in transgenic *Nicotiana benthamiana* [6,143]. In addition to the above-mentioned WRKY proteins, several members of this family identified from other plants, such as *RtWRKY1* from *Reaumuria trigyna* [65], banana *MaWRKY26* [144], and chrysanthemum *DgWRKY5* [145], are also important components in abiotic stress signaling pathways.

### 2.4. bZIP Transcription Factors

The bZIP (basic leucine zipper) TF family, characterized by a highly conserved basic DNA-binding domain consisting of 16 basic amino acid residues and an adjacent dimerization motif known as leucine zipper, is extensively distributed in eukaryotes [146]. A lot of putative bZIP TFs have been identified at genome wide scale from several sequenced plant species and are categorized into three different groups.

In plants, members of bZIP TFs function in a diverse range of biological processes, including cell elongation, organ and tissue differentiation, primary root growth, flower development and seed maturation, plant senescence, and light response [147,148,149], as well as abiotic stress responses such as cold, high salinity, drought, and others. [150]. Especially in Arabidopsis, group A members of bZIP TFs family, including ABF1 [151], ABF2/AREB1 [152], ABF3 [153], ABF4 [154], and ABI5 [155], play important roles in ABA signal transduction and abiotic stress responses. Meanwhile, a member of group S1 bZIP factors, Arabidopsis bZIP1 has been shown to enhance salt and drought stress tolerance [156]. Hartmann et al. [157] reported that the expression of *bZIP53*, from group S1 bZIP TFs, was transcriptionally induced by salt treatment in Arabidopsis root and plays an important role in root metabolic reprogramming under salt stress. Similarly, transgenic alfalfa (*Medicago sativa*) overexpressing the Arabidopsis *ABF3* TF under the control of sweet potato (*Ipomoea batatas*) oxidative stress-inducible *SWPA2* promoter showed better growth under drought [66]. Genetic studies exhibited that *OsABF1* and its closest homolog *OsbZIP40* function redundantly to delay flowering time in rice under drought stress via indirect suppression of *Ehd1* (Early heading date 1), encoding a key activator of flowering in rice, suggesting a direct connection between plant developmental program and water availability [67]. Moreover, expression of *OsbZIP71* was repressed under NaCl treatment but, interestingly, *OsbZIP71* overexpression displayed significantly high tolerance to salt stress in addition to drought and osmotic stresses. By contrast, *OsbZIP71* RNAi lines showed sensitivity to salt and ABA treatment, suggesting a vital role of *OsbZIP71* in ABA-mediated salt and drought stress tolerance [158]. Also, a recent study revealed that *OsABF1* can enhance drought resistance in rice, probably via transcriptional activation of its target gene, *COR413-TM1*, encoding a putative thylakoid membrane protein [146]. Zong et al. [159] found that *OsbZIP23* is the main player in rice ABA biosynthesis and drought tolerance via directly regulating the expression of numerous stress-responsive genes, such as *OsPP2C49* and *OsNCED4*. Similarly, Dey et al. [160] identified two polymorphic forms of *OsbZIP23* gene from wild rice genotypes. Functional characterization of these variants (1083 bp and 1068 bp CDS) via overexpression and gene silencing (RNAi) established that enhanced expression of *OsbZIP23*, instead of natural polymorphism in its coding sequence, is responsible for drought tolerance and improved grain yield in rice. Very recently, co-overexpression of the constitutively active form of *OsbZIP46*, along with a protein kinase (SAPK6), resulted in significantly high drought resistance [68]. Similarly, in wheat, functional analysis of a *TaABL1* protein in transgenic Arabidopsis and tobacco showed that this gene is involved in accelerating stomatal closure and accumulation of osmotic substances under different stress conditions, thereby improving tolerance to multiple abiotic stresses [69]. Under cold stress, the down regulated expression of CBFs and some key COR genes in *TabZIP6*-overexpressing Arabidopsis seedlings led to decreased freezing tolerance [70]. Also, *TabZIP60* plays an important function in multiple abiotic responses, however, enhancing plant sensitivity to abscisic acid (ABA) during seedling growth [71]. Introduction of a maize bZIP, *ABP9*, in cotton led to reduced stomatal aperture, better root system, and higher relative water content under multiple abiotic stress conditions [72]. In tomato, the negative role of *SlbZIP38* in drought and salt stress was linked to higher level of MDA and lower chlorophyll and free proline content in leaves [73]. In *Eleusine coracana*, transcripts of *EcbZIP60* were highly induced by salt-, drought-, and methyl viologen-induced stress, and promoted abiotic stress tolerance in transgenic tobacco by activating the expression of unfolded protein-responsive pathway genes, including *PDIL*, *CRT1*, and *mBiP1* [74]. Functional analysis of *GmbZIP110* from soybean revealed that it can bind to the ACGT motif and modulate the expression of many stress-related genes in transgenic Arabidopsis. In addition, transgenic soybean composite seedlings and Arabidopsis exhibited enhance salt tolerance in terms of higher proline content under salt stress, indicating that *GmbZIP110* acts as a positive regulator of salt stress tolerance [75]. Overexpression of a group A member of bZIP proteins, *GmFDL19*, significantly increased plant relative height and relative shoot dry weight at seedling stage in transgenic soybean following salt and polyethylene glycol (PEG 6000) treatments, suggesting an important role of *GmFDL19* in soybean abiotic stress responses [76]. Moon et al. [77] reported that when ectopically expressed, *CaBZ1* from hot pepper (*Capsicum annuum*) enhances dehydration tolerance in transgenic potato without any negative effect on plant growth and tuber yield. Similarly, *GhABF2* from cotton has been shown to facilitate drought and salt stress tolerance in transgenic cotton through an ABA-dependent pathway, as supported by higher antioxidant enzyme activities and proline content in transgenic plants under drought and salt stress [78]. In addition, functional analysis of grape (*Vitis vinifera*) *VvbZIP23* [161], lotus root (*Nelumbo nucifera*) *LrbZIP* [162], *T. hispida ThbZIP* [163], and ramie (*Boehmeria nivea*) *BnbZIP2* [79] genes demonstrated that all these bZIP TFs play critical roles in regulating salt stress along with other abiotic stresses.

### 2.5. NAC (NAM, ATAF, and CUC) Transcription Factors

The NAC (NAM, ATAF, and CUC) proteins originally reported from petunia (*Petunia hybrida*) NAM [164] and Arabidopsis [165] form one of plant-specific transcription factor families. Typically, NAC family proteins are characterized by a well-conserved DNA-binding NAC domain within the N-terminal and a highly variable transcription regulatory domain in the C-terminal, which might function as transcriptional activators or repressors to regulate the expression of downstream target genes [166,167].

The NAC TF family widely occurs in plants, and is multifunctional in plant growth and development, as well as in responses to abiotic stress [168]. For instance, *JUB1* (JUNGBRUNNEN1), a NAC protein from Arabidopsis, was recently shown to promote dehydration tolerance under the control of both RD29A (abiotic stress-induced) and CaMV 35S (constitutive) promoters [81]. Another NAC TF gene, *ATAF1*, was induced by ABA treatment and high salinity. *ATAF1* transgenic rice exhibited salt tolerance and insensitivity to ABA [169]. By contrast, *AtNAP* negatively participates in the salt stress response during seed germination and plant development via transcriptional repression of the ABA-dependent pathway-related genes (*AREB1*, *RD20*, and *RD29B*) [170]. Also, *ANAC069*-overexpressing plants displayed decreased proline biosynthesis and ROS-scavenging capability, which resulted in increased sensitivity to salt and osmotic stress [82]. Also, transcripts of *GsNAC019* were upregulated in the roots of *Glycine soja* by less than 50 mM NaHCO_3_ conditions, and its overexpression in Arabidopsis conferred tolerance to alkaline stress during seedling and mature stages but led to reduced ABA sensitivity [83]. In rice, *SNAC3* was found to modulate homeostasis of reactive oxygen species (ROS), thereby enhancing heat and drought stress tolerance [171]. *ONAC022* overexpression in rice resulted in higher survival ratios and accumulated less Na^+^ in roots and shoots in response to drought and salt stress, respectively [84]. Likewise, at flowering stage, *OsNAP*-overexpressing plants exhibited better yield under drought stress, in addition to enhanced tolerance against multiple abiotic stresses at the vegetative stage, without any growth retardation [172]. In wheat, under the control of a predominantly root-expressed promoter, *TaRNAC1* enhanced dehydration tolerance in terms of higher biomass, grain yield, and root length [85]. Likewise, overexpression of *TaNAC47* enabled plants to withstand salt, drought, and freezing stresses [86]. Furthermore, *CarNAC4*—a putative stress-associated TF from chickpea (*Cicer arietinum*)—was found to be involved in reducing water loss rates and MDA content in response to drought and salt stress, respectively [173]. *MfNACsa*, a lipid-anchored NAC gene from *Medicago falcata*, positively regulates plant drought stress tolerance through differential expression of oxidation–reduction-related, lipid transport-related, and localization-related genes [87]. In tomato, virus-induced gene silencing of *SlJUB1* led to drastic reduction in plant drought tolerance, compared to control plants, and highly promoted oxidative stress [174]. Also, *SlNAC4*-RNAi plants exhibited a higher water loss rate and lower chlorophyll content under drought and salt stress [88]. Similarly, *SlNAC35* is involved in promoting root growth and development under salt and drought stress in transgenic tobacco [89]. Likewise, the improved abiotic stress resistance in *MlNAC9*-overexpressing plants is primarily attributed to the enhanced capability to scavenge reactive oxygen species (ROS) and increased expression of stress-responsive genes through an ABA-dependent pathway [175]. In addition, sorghum *SbSNAC1* [176], maize *ZmNAC55* [90], and wheat *TaNAC29* [177] also play positive roles in plant drought and/or salt stress tolerance. On the other hand, *TaNAC2L* [91] from wheat and *SlNAM1* [92] from tomato were reported to play important roles in heat and cold stress, respectively. By contrast, functional analysis through dominant chimeric repressor-mediated suppression revealed that *ONAC095* performs opposite roles during drought and cold stress conditions in rice by acting as a positive regulator of cold response and a negative regulator of drought response [178]. Recently, An et al. [179] reported for the first time that overexpression of *MdNAC029*, both in apple and Arabidopsis, reduces cold tolerance through the CBF-dependent pathway. Functional characterization of *Pyrus betulifolia* NAC TF, *PbeNAC1*, showed that this gene is involved in mediating drought and cold tolerance [80]. *MusaNAC042*-overexpressing lines of banana demonstrated high chlorophyll and lower MDA content in response to high salinity and dehydration stress, suggesting a positive role of *MusaNAC042* in abiotic stress [180].

### 2.6. Other Transcription Factors

Several TFs from other TF families, including AP2/ERFs (APETALA2/ethylene response factor), CAMTA (calmodulin binding transcription activator), cycling Dof factor (CDF), and C_2_H_2_-ZFP, have been reported to participate in abiotic stress responses in a variety of plants. Ectopic expression of the Arabidopsis *DREB1A/CBF3* gene displayed higher photosynthetic rate and antioxidant activities in transgenic *Salvia miltiorrhiza* plants under drought stress [181]. Recently, Ahn et al. [182] uncovered the molecular mechanisms of dehydration tolerance in “Nipponbare”, a transgenic version of rice (erf71) carrying *OsERF71*, and showed that erf71 strongly resists drought stress. Also, ERF6 (ETHYLENE RESPONSE FACTOR6) protein has been positively linked to ROS-signaling during plant growth, as well as biotic and abiotic stress tolerance in Arabidopsis [183]. Similarly, in Arabidopsis, members of AP2 TF family, including *DREB19*, *DREB26*, *RAP2.6*, and *RAP2.6L*, were shown to participate positively in regulating plants in response to abiotic stresses [184]. Transgenic tobacco plants overexpressing *SbDREB2A* from *Salicornia brachiata* displayed improved seed germination and growth characteristics under hyperosmotic and hyperionic stresses [185]. Likewise, SsDREB protein cloned from *Suaeda salsa* promoted a net photosynthesis rate in transgenic tobacco under salt and drought stress [186]. Further, *CAMTA1* (calmodulin binding transcription activator) TF was found to be involved in regulating drought recovery in Arabidopsis [187]. Prasad et al. [188] reported that a member of signal responsive transcription factors, *SR1/CAMTA3*, decreases salt tolerance via inhibiting the expression of salt stress-related genes. In tomato, the *CDF3* gene, belonging to the cycling Dof factor (CDF) TF family, was found to be associated with higher yield and biomass production under salt stress [189]. Additionally, overexpression of *TAZAT8*, a member of the C2H2-ZFP family from wheat, conferred tolerance to Pi-starvation stress in tobacco by regulating ROS detoxicity and Pi acquisition [190].

## 3. Potential Molecular Mechanisms of TFs in Controlling Plant Abiotic Stress Responses

### 3.1. TFs Regulating ROS Signal Transduction during Abiotic Stress Responses in Plants

It is well known that when plant cells perceive abiotic stress stimulus via receptors or sensors, ROS accumulation significantly increases, which results in cell oxidative damage and eventually causes cell death. Recently, ROSs have been also identified as key second messengers in the complex signaling network of plant abiotic stress responses. Therefore, regulating ROS signaling and its homeostasis is an important strategy to improve stress tolerance of plants suffering from unfavorable environmental conditions [191]. As one kind of the important regulatory proteins involved in abiotic stress responses, TFs play vital roles downstream of ROS signaling pathways. Members of MYB, bHLH, WRKY, bZIP, and NAC families have been demonstrated to play important roles in regulating ROS signal transduction during abiotic stress responses in plants. For example, Mabuchi et al. reported the ROS-responsive Arabidopsis *MYB30* plays a key role in regulating root growth during ROS defense responses [192]. GmMYB84, another MYB TF from soybean, also plays important roles in ROS homeostasis regulation and abiotic stress resistance in plants. Expression of *GmMYB84* is induced by drought, salt, H_2_O_2_, and ABA. Overexpression of *GmMYB84* enhanced transgenic soybean drought tolerance with higher ROS content and antioxidant enzyme activities (superoxide dismutase (SOD), peroxidase (POD), and catalase (CAT)) [30]. Ectopic expression of *TaMYB33* exhibits greater ability for ROS detoxification and reconstruction of osmotic balance in Arabidopsis due to induction of *AtZAT12* and *AtP5CS* genes, responsible for replicating the activities of ascorbate peroxidase and proline synthesis, respectively [98]. Recently, an R2R3-MYB gene, *TaODORANT1*, cloned from wheat was upregulated by H_2_O_2_ treatment, and facilitated ROS scavenging in transgenic tobacco via regulating CAT and SOD activities under salt and drought stress, respectively [193]. There is growing evidence that bHLH TFs facilitate ROS detoxification under abiotic stress conditions. For instance, functional characterization of *NtbHLH123*, a bHLH TF gene, revealed that this TF confers cold tolerance to *NtbHLH123*-overexpressing transgenic tobacco by regulating the NtCBF (C-repeat biding factor) pathway and ROS homeostasis [194]. Similarly, Geng and Liu demonstrated CsbHLH18 of sweet orange (*Citrus sinenisis*) also functions in modulation of cold tolerance and ROS homeostasis via regulating the antioxidant gene *CsPOD* [195]. Also, gain and loss of function analysis revealed that *Tamarix hispida* ThbHLH1 TF positively controls ROS detoxification and enhances this second messenger to mediate abiotic stress tolerance [52]. WRKY proteins govern numerous ROS-dependent abiotic stress responses through activation of the cellular antioxidant systems [57,61,64]. In groundnut (*Arachis hypogaea*), overexpression of *MuWRKY3* promoted ROS detoxification, accompanied by increased activities of antioxidant enzymes under drought stress [196]. Similarly, Hong et al. [197] reported that maize *ZmWRKY4* is required for upregulated expression and activities of ZmSOD4 and ZmcAPX under cadmium stress, indicating that *ZmWRKY4* plays a vital role in controlling antioxidant defense following stress. Currently, it has been revealed by a number of research reports that NAC proteins are critically involved in escalating the antioxidant defense system under abiotic stresses. In cucumber (*Cucumis sativus*), CsATAF1 was recently shown to promote ROS detoxification via direct regulation of three antioxidant system genes, including *CsCu-ZnSOD*, *CsABI5*, and *CsDREB2C*, improving tolerance to drought stress [198]. On the other hand, TaNAC29 from wheat was reported to reduce membrane damage and H_2_O_2_ accumulation by enhancing the antioxidant system in plants, which finally culminates in salt stress tolerance [177].

### 3.2. TFs Binding with cis-Element in the Promoters of Stress-Inducible Genes

Transcription factors play a vital role in plant adaptation to environmental cues because of their ability to control critical downstream responses via regulating target gene expression. Entire signal transduction cascades are activated when TFs bind to diverse TF binding sites (TFBS) in the promoters of stress-inducible genes [20,21,22,23,199]. Therefore, a comprehensive understanding of underlying mechanisms of transcription factors and their targeted stress-inducible genes has become imperative to unravel their functionality in various abiotic stress responses. Recently, Wang et al. [30] dissected the mechanism of *GmMYB84* in promoting root growth in soybean both under optimal and drought conditions and showed that overexpression of *GmMYB84* results in primary root elongation under drought stress via directly binding to the MBSI motifs in the promoters of *GmRBOHB-1* and *GmRBOHB-2* genes, which in turn generate higher levels of H_2_O_2_. The rise in ROS concentration then activates the expression of genes of antioxidant enzymes SOD, CAT, and POD to scavenge ROS. In *M. truncatula*, Zhang et al. [108] uncovered the transcriptional regulation of C-repeat binding factor, *MtCBF4*, under cold stress, and found that the expression of *MtCBF4* is inhibited by *MtMYB3* protein via binding to its promoter. Interestingly, this inhibitory effect of *MtMYB3* on *MtCBF4* is antagonistically relieved by *MtMYB61*, resulting in improved freezing tolerance and cold acclimation. It was shown that wheat TaSIM, an R2R3-MYB TF, promotes salt tolerance in Arabidopsis via binding to the MBS II motifs in the promoters of *RD22* and *RD29A* genes, indicating that TaSIM is an upstream activator of these genes, and facilitates crosstalk between ABA-dependent and ABA-independent pathways [36]. Lee et al. [29] demonstrated that MYB96, an R2R3-type MYB protein, is responsible for integrating the ABA and cold signaling pathways in Arabidopsis by binding to the promoters of *HHP* (HEPTAHELICAL PROTEIN) genes, consecutively interacting with upstream CBF regulators such as *ICE1*, *ICE2*, and *CAMTA3*, thus facilitating their transcriptional activation in response to stressful conditions. Very recently, *MdMYB88* and *MdMYB124* were found to modulate the expression of cold-responsive genes *MdCSP3* and *MdCCA1* through binding to the AACCG motifs in their promoters under cold stress and increase cold hardiness both in apple and Arabidopsis [41]. Wei et al. [112] revealed that chicory *CiMYB3* and *CiMYB5* proteins contribute to the orchestration of fructan degradation under abiotic stress and hormonal treatments by recognizing the MYB-core motifs (C/TNGTTA/G) in the promoters of fructan 1-exohydrolase (*1-FEH*) genes. Similarly, *OsMYBR1* was found to contain a cis-acting sequence, ABRE, in its promoter, and to confer dehydration tolerance to transgenic Arabidopsis via induction of stress-responsive genes such as *OsProt*, *OsLEA3*, *OsP5CS*, and *OsRab16* [103]. Yeast one-hybrid screening showed that *OsPIF14*, a putative phytochrome-interacting bHLH TF, binds to the promoter of cold-responsive *OsDREB1B TF* thereby represses its expression through an extended N-box, suggesting their involvement in crosstalk between light and stress signaling [200]. In Arabidopsis, WRKY71 functions antagonistically against salt-induced late flowering by regulating *LFY* (LEAFY) and *FT* (FLOWERING LOCUS T) genes [56]. Under salt stress, *RtWRKY1* increases the expression of proline biosynthesis (*AtP5CS*1 and *AtP5CS2*) genes in WT, and *AtSOS*1 in transgenics, which in turn regulate Na^+^ extrusion in response to salt stress, indicating that *RtWRKY1* promotes the osmoregulatory capacity of transgenics via induction of Na^+^ extrusion and proline biosynthesis [65]. Zong et al. [159] reported that *OsbZIP23* is transcriptionally activated via interacting with *SAPK2* (Suc nonfermenting-1-related protein kinase2), which in turn interacts with an ABI1 homolog, *OsPP2C49*, resulting in the inhibition of *OsbZIP23* activity. Further, *OsbZIP23* can directly regulate *OsNCED4* (ABA biosynthesis) and *OsPP2C49* genes, suggesting that *OsbZIP23* is involved in feedback regulation of ABA biosynthesis and signaling, as well as drought tolerance, in rice. TabZIP6 protein negatively regulates freezing tolerance through the downregulated expression of *CBF1*, *CBF3*, and some *COR* genes in transgenic Arabidopsis [70]. *VaNAC26* from *Vitis amurensis* was reported to participate in transcriptional regulation of JA (jasmonic acid) synthesis, and signaling genes including *AOC1*, *LOX2*, *PDF1.2*, *VSP1*, and *MYC2* via binding to NACRS motifs in their promoters thus impart drought tolerance [93]. In Arabidopsis, *ANAC069* inhibited the expression of proline biosynthesis (*P5CS*) and antioxidant (*POD*, *SOD*, and *GST*) genes under salt stress via binding to C[A/G]CG[T/G] sequences in their promoters, which led to enhanced salt and osmotic stress sensitivity [82]. *MdNAC029*, cloned from apple, binds to the promoters of *MdCBF1* and *MdCBF4* genes, thereby repressing their transcript levels that lead to reduced cold tolerance [179].

### 3.3. The Significance of Protein–Protein Interactions

**Figure 2:**
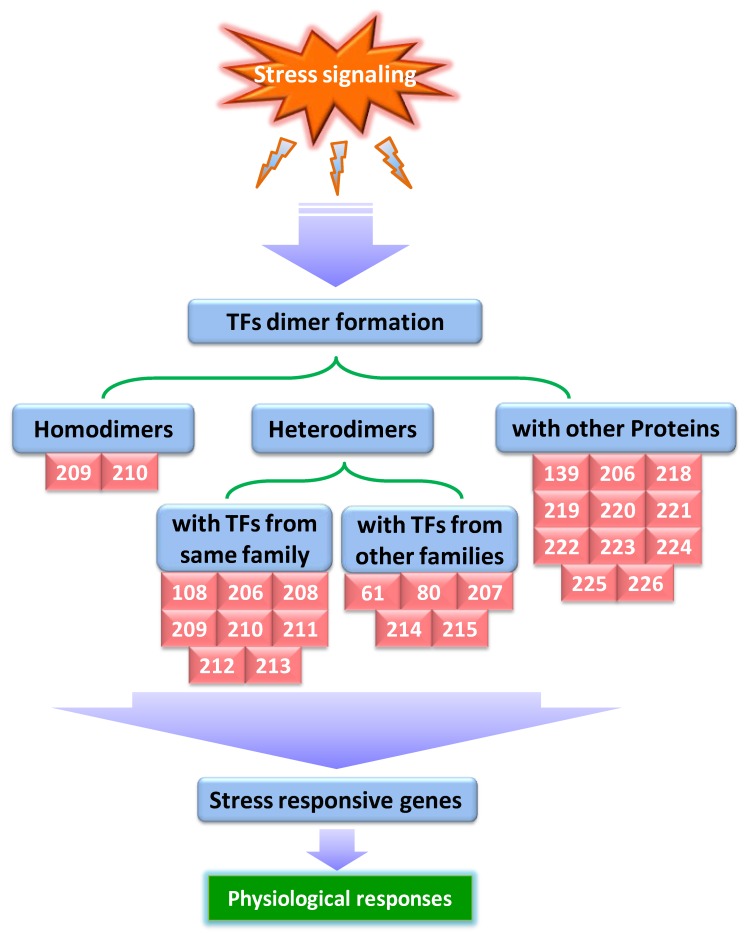
Model for dimer formation of transcription factors during abiotic stress responses. Numbers in red square boxes represent corresponding references.

Recent developments in molecular biology have shown that proteins very often establish combinatorial interactions with each other, rather than acting alone, to carry out their complex biological functions [201]. In general, the term “protein–protein interactions” refers to the association of two or more protein molecules binding together and comprises a range of events, like functional and physical interactions, as well as transient and permanent complexes [202] (Figure 2). The coordination of protein–protein interactions (PPIs) mostly mediates the overall molecular architecture, both structurally and functionally in all organisms. The majority of cellular processes is dictated by protein–protein interactions and is thought to participate in governing responses of organisms under different environments, including stress conditions (Figure 2).

It has been well established that a significant level of crosstalk exists between the signal transduction pathways during abiotic stress conditions involving two or more TFs [203]. A bulk of TFs can form homodimers or heterodimers through physical interactions with an identical protein or other members of the same family, respectively, or establish complexes with TFs from other protein families [204], offering huge combinatorial flexibility during the regulation of transcription [205]. In plants, intra- and interfamily TF interactions have been widely studied during the last decades. Here, we discuss the recent understandings of TF interactions and their functions during abiotic stress tolerance. Using yeast two-hybrid (Y2H) and bimolecular fluorescence complementation (BiFC) assays, Zhang et al. [108] reported that MtMYB3 interacts with MtMYB61 in *M. truncatula* and this interaction in turn results in relieving the inhibitory effect of MtMYB3 on the expression of a C-repeat binding factor, *MtCBF4*, conferring freezing tolerance. Likewise, stress-responsive MYB TFs, MYBR1 and MYBR2, physically interact with each other and are partially redundant in their functions, as indicated by senescence and stress-related phenotypes of double mutant *mybr1mybr2* that are stronger than single *mybr1* and *mybr2* mutants [206]. Also, in soybean, the interaction of GmMYB174 with GmWRKY27 protein suppresses the expression of a downstream target gene, *GmNAC29*, which acts as a negative effector of stress tolerance, conferring drought stress tolerance [61]. Deng et al. [207] revealed that physical interaction of two basic helix-loop-helix TFs, OsICE1 and OsICE2, with OsMYBS3 TF, promotes cold tolerance in rice. Also according to Feng et al. [208], physical interaction between *O11*, a bHLH TF, and ZmICE1 TF in maize leads to the integration of stress responses and endosperm development. In recent times, Wang et al. [209] demonstrated that ThWRKY4 from *Tamarix hispida* is a dimeric protein that can form both homodimers via self-interaction and heterodimers with ThWRKY2 and ThWRKY3 to regulate abiotic stress responses. Similarly, two other WRKY proteins from walnut (*Juglans regia*), JrWRKY2 and JrWRKY7, both were found to form homodimers and can also interact with each other to mediate seed development and abiotic stress responses [210]. Additionally, functional interactions among WRKY25, WRKY26, and WRKY33 proteins promote heat stress tolerance in Arabidopsis via cooperative regulation of heat shock proteins related and ethylene-activated signaling pathways [211]. Further, yeast two-hybrid experiments confirmed that Arabidopsis WRKY6 can interact with WRKY42, and both proteins act in plant Pi-deficient environments by repressing the transcription of *PHO1* (PHOSPHATE1) via binding to W-box motifs in its promoter [212]. In wheat, co-expression of drought-responsive γ-clade HD-Zip I TF genes, *TaHDZipI-3*, *TaHDZipI-4*, and *TaHDZipI-5*, in the same tissue, in addition to their ability to heterodimerize, suggests their role in wheat response to water deficit [213]. PbeNAC1, cloned from *Pyrus betulifolia*, enhances the transcript levels of stress-responsive genes through physical interaction with PbeDREB1 and PbeDREB2A TF proteins, thereby mediating cold and drought stress tolerance in transgenic tobacco [80]. In peanut (*Arachis hypogaea*), the *AhAREB1*/*AhNAC2* protein complex leads to inhibition of *AhNCED1* expression under drought stress and acts as a negative feedback regulator of ABA biosynthesis [214]. Likewise, Shan et al. [215] described that a cold-inducible *MaNAC1* TF from banana is not only a direct target of *MaICE1*, but also physically interacts with MaCBF1, indicating that MaNAC1 regulates cold stress tolerance through its interaction with ICE1-CBF cold-signaling pathway. Overall, these studies provide helpful insights into TF interaction events and define their roles in plant abiotic stress responses.

Functional modulation of transcription factors via interactions with regulatory proteins is an important step in the regulation of signal transduction pathways [216,217]. Recently, several research studies have established that interactions between TFs with their interacting partners (proteins) have a significant role in mediating abiotic stress tolerance in plants [218,219]. For example, in chickpea, two putative transcription factors, Myb.Ph and Athb-1, bind to the promoter of a stress-responsive ferritin protein, CaFer1; in addition, CaFer1 interacts with iron transporter IRT1, and the resulting ferritin1-complex then mediates iron homeostasis and drought stress tolerance [218]. A *CCA1*-like MYB protein, GmMYB138a, interacts with GmSGF14l (a 14-3-3 protein) that alters the subcellular localization of GmMYB138a. qPCR analysis demonstrated that GmMYB138a and GmSGF14l respond synergistically or antagonistically to salt, drought, and cold stresses [220]. In addition, Jaradat et al. [206] stated that Arabidopsis MYBR1 physically interacts with an ABA receptor protein, PYL8, and directly participates in early ABA signaling. Moreover, Li et al. [221] also identified AtMYB44 as an interacting partner of RCAR1/PYL9 protein, an ABA receptor, and showed that this interaction reduces the inhibitory effect of RCAR1/PYL9 on ABI1 activity, implying that AtMYB44 negatively regulates ABA signaling. Using yeast two hybridization and CoIP approaches, recently, Zhou et al. [222] screened out a small ubiquitin-like modifier E3 ligase MdSIZ1 as an interacting protein of MdMYB1 that enhances anthocyanin accumulation in apple under low temperature via sumoylation of MdMYB1 protein. Using both GST-pulldown and BiFC assays, Wang et al. [223] disclosed that CgbHLH001 from *Chenopodium glaucum* is a potential interaction component of CgCDPK protein in the signal transduction pathway in response to drought or salt stress. In Arabidopsis, the interaction of WRKY8, a positive regulator of salt stress, with VQ9 (a VQ motif-containing protein) in the nucleus, decreases the DNA-binding activity of WRKY8, resulting in modulation of salt stress tolerance [224]. Y2H assay revealed that direct interaction of rice OsWRKY30 with OsMPK3 protein causes phosphorylation of OsWRKY30, leading to improved drought tolerance [139]. In rice, interaction of MODD (Mediator of OsbZIP46 deactivation and degradation, a homolog of AFP) protein with OsbZIP46 through the D domain upsets the regulation of ABA signaling and drought tolerance [225]. Using the Y2H system, Vivek et al. [219] identified NAC TF as an interacting partner of ZoCDPK1 (*Zingiber officinale* Calcium-dependent protein kinase 1) and showed that ZoCDPK1 operates in ginger through NAC-mediated, ABA-independent, cold nonresponsive stress signaling pathway. Similarly, the physical interaction of late embryogenesis abundant (LEA) protein, GmLEA3.1, with soybean GmDi19-5, a member of zinc finger TF family, enhances the stability of the GmDi19-5 protein and promotes abiotic stress sensitivity in transgenic Arabidopsis lines [226].

These studies indicate that protein–protein interactions play vital roles in abiotic stress responses and offer critical understanding of the regulation of abiotic stress responses. Particularly, this approach is helpful in crop species with narrow mutant stocks, in which it is hard to set up gene interaction relationships via epistasis. Further, it can recognize proteins that might not be detected by changes in their mRNA transcript levels, in addition to the direct interaction partners of regulatory proteins [227]. Hence, protein–protein interaction studies provide a novel perspective to perceive their role in a cell and to understand molecular mechanisms that trigger appropriate plant responses under abiotic stress.

## 4. Conclusion and Future Perspectives

Abiotic stress signal cascades engage different TFs to regulate the expression of stress-responsive genes, thereby imparting stress tolerance to plants. The current research progress on the involvement of TFs in conferring abiotic stress tolerance under stress conditions highlight their fundamental job in plant growth and development. This review about the TF families discussed above indicates that plenty of transcription factors play important roles in abiotic stress tolerance, whose applications are likely to make a great improvement in crop breeding. However, understanding their functions and interactions is still insufficient and there are some difficulties ahead which must be overcome for the effective implementation of manipulating TFs to genetic engineering breeding. Firstly, functional redundancy among some TFs makes it difficult to notice mutations in single mutants. Nevertheless, the functional redundancy genes should be knocked out at the same time using the new high-efficient technique, CRISPR-Cas9-mediated genome editing [228]. Likewise, reverse-genetics approaches are also useful for breaking through this problem. For example, the creation of dominant negative mutants via binding repressor domains to transcription factors seems to be an alternative option [229]. Secondly, plants are simultaneously subjected to different abiotic stress conditions in nature. In order to survive in such environments, multiple genes and different stress response pathways are co-activated [230], resulting most likely in a synergistic or antagonistic effect on each other. In addition, owing to the complexity of regulating networks among different TFs at different levels, overexpression of certain transcription factors might affect other signaling pathways. For example, in an Arabidopsis mutant, overexpression of *AtMYB96* promotes dehydration tolerance but significantly reduces lateral root formation, probably due to multiple drought stress-signaling pathways and/or functional redundancy of multiple MYB proteins controlling lateral root formation [231]. Therefore, in future, a comprehensive understanding of regulatory networks and complete elucidation of transcription factors is direly needed if we wish to use regulatory genes as a molecular leverage to raise multiple stress-resistant crop varieties. Thirdly, TF research should be primarily focused on the identification and characterization of multiple stresses-responsive TF genes. Moreover, we should also examine the molecular effects of overexpressing TFs, in addition to conducting stress tolerance assays. In some cases, the constitutive overexpression of TF genes, although endowing transgenic plants with stress tolerance, results in growth and development defects, such as growth retardation, leaf senescence, and lower yields. This problem should be overcome through screening and applying of tissue-specific promoters, such as an epidermis-specific *CER6* promoter [232] and stress-inducible promoters like *rd29A* [233]. Additionally, despite the many studies on glycophytes, such as Arabidopsis, rice, and wheat, knowledge about xerophytes and halophytes is considerably limited. Given that xerophytic and halophytic species living in arid and saline regions contain abundant stress-resistant genes and have evolved specialized strategies to survive in harsh environments [234,235], to identify these key genes and understand the functional mechanisms of the naturally tolerant species will contribute to developing stress tolerance in crops. In conclusion, although genome-wide analysis has identified a great deal of TFs associated with stress tolerance, nevertheless, their functional validity, crosstalk, and interactions during abiotic stress require further, deeper understanding. In-depth knowledge about a large number of TFs and their downstream target genes can provide better elucidation of regulatory mechanisms involved in plant abiotic stress tolerance progress, which supports acquiring ideal candidate TF genes and developing strategies for generating stress-tolerant crops with higher qualities and yields.

## Figures and Tables

**Table 1:** Transcription factors involved in regulating plants in response to abiotic stresses.

Gene Family	Gene	Identified in Crop	Studied Crop	Stress	References
**MYB**	*FtMYB9*	*Fagopyrum tataricum*	Arabidopsis	Drought/Salt	[24]
	*1R-MYB*	*Cicer arietinum*	Chickpea	Drought	[28]
	*MYB96*	*Arabidopsis thaliana*	Arabidopsis	Cold	[29]
	*GmMYB84*	*Glycine max*	Soybean	Drought	[30]
	*GmMYB12B2*	*Glycine max*	Arabidopsis	Salt	[31]
	*OsMYB55*	*Oryza sativa*	Maize	Drought	[32]
	*OsMYBc*	*Oryza sativa*	Rice	Salt	[33]
	*MID1*	*Oryza sativa*	Rice	Drought	[34]
	*OsMYB91*	*Oryza sativa*	Rice	Salt	[35]
	*TaSIM*	*Triticum aestivum*	Arabidopsis	Salt	[36]
	*MtMYBS1*	*Medicago truncatula*	Arabidopsis	Salinity	[37]
	*ARS1*	*Solanum lycopersicum*	Tomato	Salt	[38]
	*GaMYB62L*	*Gossypium arboreum*	Arabidopsis	Drought	[39]
	*GaMYB85*	*Gossypium arboreum*	Arabidopsis	Drought	[40]
	*MdMYB88/MdMYB124*	*Malus domestica*	Arabidopsis/apple	Cold	[41]
	*PacMYBA*	*Prunus avium*	Arabidopsis	Salt	[42]
**bHLH**	*AtbHLH68*	*Arabidopsis thaliana*	Arabidopsis	Drought	[43]
	*PebHLH35*	*Populus euphratica*	Arabidopsis	Drought	[44]
	*AtbHLH112*	*Arabidopsis thaliana*	Arabidopsis	Salt/Drought	[45]
	*OsbHLH068*	*Oryza sativa*	Arabidopsis	Salt	[46]
	*TabHLH39*	*Triticum aestivum*	Arabidopsis	Drought/Salt/Cold	[47]
	*FtbHLH2*	*Fagopyrum tataricum*	Arabidopsis	Cold	[48]
	*FtbHLH3*	*Fagopyrum tataricum*	Arabidopsis	Drought/Oxidative	[49]
	*AH*	*Solanum lycopersicum*	Tomato	Low-temperature	[50]
	*PubHLH1*	*Pyrus ussuriensis*	Tobacco	Cold	[51]
	*ThbHLH1*	*Tamarix hispida*	Arabidopsis	Osmotic stress	[52]
	*TabHLH1*	*Triticum aestivum*	Tobacco	Pi/N-starvation	[53]
	*PtFIT*	*Populus tremula*	Populus	Iron	[54]
**WRKY**	*OsWRKY47*	*Oryza sativa*	Rice	Drought	[55]
	*WRKY71*	*Arabidopsis thaliana*	Arabidopsis	Salt	[56]
	*AtWRKY53*	*Arabidopsis thaliana*	Arabidopsis	Drought	[57]
	*ZmWRKY17*	*Zea mays*	Arabidopsis	Drought	[58]
	*OsWRKY71*	*Oryza sativa*	Rice	Cold	[59]
	*TaWRKY146*	*Triticum aestivum*	Arabidopsis	Drought	[60]
	*GmWRKY27*	*Glycine max*	Soybean	Drought/Salt	[61]
	*SlWRKY3*	*Solanum lycopersicum*	Tomato	Salt	[62]
	*MtWRKY76*	*Medicago truncatula*	Medicago	Salt/Drought	[63]
	*GhWRKY6-like*	*Gossypium hirsutum*	Arabidopsis	Salt	[64]
	*RtWRKY1*	*Reaumuria trigyna*	Arabidopsis	Salt	[65]
**bZIP**	*AtABF3*	*Arabidopsis thaliana*	Alfalfa	Drought/Salt	[66]
	*OsABF1*	*Oryza sativa*	Rice	Drought	[67]
	*OsbZIP46*	*Oryza sativa*	Rice	Drought/Temperature	[68]
	*TaABL1*	*Triticum aestivum*	Arabidopsis	Cold/Drought/Salt	[69]
	*TabZIP6*	*Triticum aestivum*	Arabidopsis	Freezing	[70]
	*TabZIP60*	*Triticum aestivum*	Arabidopsis	Cold/Drought/Salt	[71]
	*ABP9*	*Zea mays*	Cotton	Salt/Drought	[72]
	*SlbZIP38*	*Solanum lycopersicum*	Tomato	Salt/Drought	[73]
	*EcbZIP60*	*Eleusine coracana*	Tobacco	Drought	[74]
	*GmbZIP110*	*Glycine max*	Arabidopsis	Salinity stress	[75]
	*GmFDL19*	*Glycine max*	Soybean	Salt/Drought	[76]
	*CaBZ1*	*Capsicum annuum*	Potato	Drought	[77]
	*GhABF2*	*Gossypium hirsutum*	Arabidopsis	Drought	[78]
	*BnbZIP2*	*Boehmeria nivea*	Arabidopsis	Drought/Salinity	[79]
**NAC**	*PbeNAC1*	*Pyrus betulifolia*	Tobacco	Cold/Drought	[80]
	*JUB1*	*Arabidopsis thaliana*	Arabidopsis	Dehydration	[81]
	*ANAC069*	*Arabidopsis thaliana*	Arabidopsis	Salt/Osmotic	[82]
	*GsNAC019*	*Glycine soja*	Arabidopsis	Alkaline	[83]
	*ONAC022*	*Oryza sativa*	Rice	Salt/Drought	[84]
	*TaRNAC1*	*Triticum aestivum*	Wheat	Drought	[85]
	*TaNAC47*	*Triticum aestivum*	Arabidopsis	Salt/Drought/Freezing	[86]
	*MfNACsa*	*Medicago falcata*	Medicago	Drought	[87]
	*SlNAC4*	*Solanum lycopersicum*	Tomato	Salt/Drought	[88]
	*SlNAC35*	*Solanum lycopersicum*	Tobacco	Salt/Drought	[89]
	*ZmNAC55*	*Zea mays*	Arabidopsis	Drought	[90]
	*TaNAC2L*	*Triticum aestivum*	Arabidopsis	Heat	[91]
	*SlNAM1*	*Solanum lycopersicum*	Tobacco	Cold	[92]
	*NAC26*	*Vitis amurensis*	Arabidopsis	Drought	[93]
